# Interprofessional Training on Substance Misuse and Addiction: A Longitudinal Assessment of a Brazilian Experience

**DOI:** 10.3390/ijerph20021478

**Published:** 2023-01-13

**Authors:** Liz Paola Domingues, Elaine Lucas Dos Santos, Danilo Polverini Locatelli, André Bedendo, Ana Regina Noto

**Affiliations:** 1 Departamento de Psicobiologia, Universidade Federal de São Paulo, São Paulo 04021-001, Brazil; 2 Centro de Ciências Biológicas, Universidade Estadual do Norte do Paraná, Bandeirantes 86360-000, Brazil; 3 Department of Health Sciences, Faculty of Sciences, University of York, York YO10 5DD, UK

**Keywords:** substance use disorders, interprofessional education, assessment of outcomes, mixed methods

## Abstract

Although several trainings have been offered to improve professional expertise on alcohol and other drugs, few have used an interdisciplinary approach and evaluated long-term improvements in the professional’s work routine. This study aimed to evaluate the outcomes of an interprofessional training program on alcohol and other drugs offered by a Regional Reference Center for Drugs of the Federal University of São Paulo, Brazil. Methods: the evaluation was carried out longitudinally using mixed methods (questionnaires (n = 29–177) and semistructured interviews (n = 28)). The participants were current workers from public institutions of health, education, social assistance, justice, and security system who attended the training. Data were collected at the beginning, the end, and one year after the end of the training. Descriptive statistical analyses were performed for quantitative data and thematic content analyses for qualitative data. Results: professionals who attended the training enhanced their understanding of substance-related issues, reduced stigma, changed their attitude, and improved their networking among the different services providing care to users. The main characteristics related to these outcomes were the interprofessional and biopsychosocial approach, and the experiential activities developed during the training. Most participants reported difficulties in implementing changes in their work routine, but those in managerial roles have reported having more autonomy to carry out such changes. Conclusions: the mixed methods converged in terms of their results. The training promoted a better understanding of issues related to substance use disorders, reduced stigma and expanded the repertoire of skills. The interprofessional and biopsychosocial approach and field activities seem to be related to these outcomes. The potential for implementing changes in daily practice was prominent among those occupying a managerial role.

## 1. Introduction

The multifactorial basis and the diversity of problems related to the consumption and dependence on alcohol and other drugs (AOD) requires professionals from different areas who are trained to identify and intervene early in related cases [1]. The literature shows that technical and academic training on the subject is very weak in the various specialties involved with assisting users and their families [2,3], with no knowledge building or the development of skills related to the area [2,3].

Most of the AOD trainings offered in the previous years focused on the performance of the physicians [4,5,6]. Promoting the biopsychosocial approach on AOD through interprofessional training is known to enable the overlapping of different areas of knowledge in the construction of teamwork, enabling dialogue and collective thought to better decision-making [7,8,9]. Given the need to train human resources to work in the field of AOD [10], it is pivotal to develop interprofessional trainings to carry out coordinated actions of prevention, early identification, and appropriate interventions in cases of substance misuse and dependence [2,3,11].

Although Muzyk et al. (2020) [12] compiled interprofessional AOD training experiences offered to undergraduate health students, there are few studies reporting the experience of interdisciplinary training programs offered for professionals who are already working in this field, such as psychologists, social workers, nurses, physicians, and others. A 2013 scoping review from Broyles et al. [13] also reported that, at that time, several trainings were offered to undergraduate professionals, and those offered for interdisciplinary working teams were not necessarily offered in the format of interprofessional classes. In addition, few studies described in detail the assessment methodology and the outcomes of training in the professionals’ routine; most were quantitative reports on satisfaction with the trainings, changes in knowledge, and self-confidence in applying the new knowledge [13].

In order to meet the needs for professional training in Brazil, in 2010, Centros Regionais de Referência em drogas (CRRs) (Regional Reference Centers for Drugs) were created by the Federal Government through partnerships with public universities. Fifty-one CRRs were established in different country regions aiming to provide training on AOD to professionals working in the public health, social assistance, education, justice, and security system network [14].

The Federal University of São Paulo (UNIFESP) offered the CRR-DIMESAD-UNIFESP (CRR-DU), one of the CRRs in the city of São Paulo. The CRR-DU began by offering two interprofessional trainings, one in 2014 and the next in 2016. The trainings focused on the development, expansion, and strengthening of collaborative networks of psychosocial care services, in addition to promoting the resoluteness of existing programs, projects, and strategies for drugs use prevention, treatment, and harm reduction. Based on the evaluation of the training offered in 2014 [1], the responsible team produced a new version of the training that was offered in 2016.

As most studies on interprofessional training in the field of AOD do not focus on long-term outcomes related to the performance of professionals [13], the aim of this study was evaluate the outcomes of the 2016 training offered by CRR-DU. Using mixed methods, this article presents the participants’ perceptions about the training and its impact on their professionals practice.

## 2. Materials and Methods

### 2.1. Study Context

This study was carried out during the second edition of the CRR-DU training, between 2016 and 2017. The training offered 600 training sites to professionals working in the areas of social assistance and development, education, health, security systems, and the justice system. Participants were referred by public departments of the city of São Paulo. The training program was organized into four sequential modules of 40 h each, and the participants could take as many of the modules as they were interested in. The first two modules, called “Contexts” and “Expertise”, used a theoretical and practical approach in the classroom, covering themes such as the contexts of substance use and dependence, and the development of specific skills for working in the area. The other two modules, “Networks and Projects” and “Technical and skills training”, used an experiential approach developed in the field, covering themes related to the development of networks, community projects, and community treatment. The modules were delivered by specialized teaching staff and comprised five to eight weekly activities, lasting eight hours each.

### 2.2. Study Design

**Figure 1:**
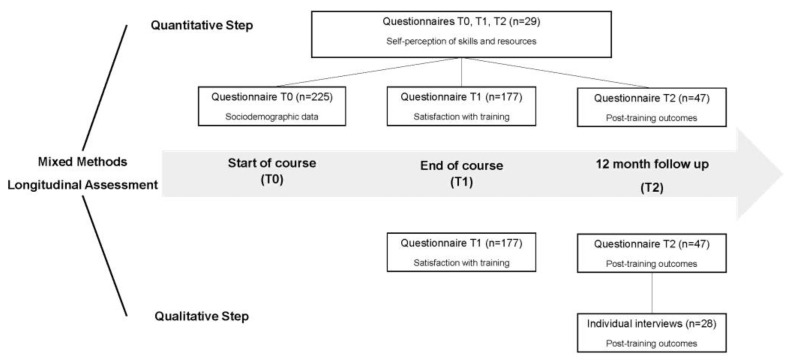
Flow of study design.

The evaluation of the training used mixed methods research [15,16] and was carried out longitudinally, with data collection points on the first day of the trainings (T0), on the last day of the trainings (T1), and one year after the end of the trainings (T2) (Figure 1). All data were collected by the research team who were properly trained and experienced in this role. Data were reported according to the GRAMMS quality criteria for mixed methods [16].

The quantitative approach included structured questionnaires completed by the training participants at T0, T1, and T2, which sought to identify objective elements in respect to the training evaluation and changes in the patterns of responses from the participants over time. The qualitative approach included open questions from the T1 and T2 questionnaires and semistructured individual interviews carried out with the participants. Individual interviews sought to deepen the participants’ perceptions about their experiences [17].

### 2.3. Data Collection

#### 2.3.1. Participants

Professionals from managerial and operational staff completing at least one of the modules offered at CRR-DU.

Pre- and post-training (T0 and T1): All training participants present on the first (T0) and last day (T1) of the first two modules were asked to complete the questionnaires in person. 

Follow up (T2): One year after the completion of the training, all participants were invited via email to complete the T2 questionnaire made available online through the REDCap platform. Reminders were sent by text message (SMS) three weeks later. Only participants who completed at least one of the modules of the training (n = 271) were invited to participate in the individual post-training follow-up interviews. These training participants were selected by drawing lots from a stratified sample, with each module defined as a stratum. The selection was carried out using an online application and invitations to participate were sent by email. We selected 51 potential participants, and the interviews were collected until we reached the theoretical saturation criterion, which occurred with 28 participants. 

The participants who took part in the individual interviews were also able to complete the T2 questionnaire before the interview. This strategy of using different routes for administering questionnaires (mixed-mode) is suggested by some authors as a strategy to increase the response rate to primarily online surveys [18,19].

#### 2.3.2. Techniques and Instruments

- Questionnaires

To protect the identity of the participants but allow the comparison of responses over time, all questionnaires were identified using a unique numeric code for each participant. Each questionnaire had open and closed self-completed questions specific to each cross-sectional analysis. The T0 questionnaire contained questions about sociodemographic data, whereas the T1 questionnaire assessed participants’ satisfaction with the training, their professional practices, and their motivation to disseminate knowledge. Finally, the T2 questionnaire sought to identify factors related to implementing knowledge in participants’ daily working routine. 

All questionnaires also contained questions about the self-perception of participants’ skills and resources to help the target population of their service relating to AOD (T0, T1, and T2). The questionnaire contained 17 items randomly distributed in three areas: 1. access to information and resources; 2. motivation and confidence for professional performance; and 3. building rapport and communication. Questions were presented as a 5-alternatives Likert scale (strongly disagree, disagree, neither agree or disagree, agree, and strongly agree).

- Individual interviews

Semistructured individual interviews were conducted one year after the end of the training, aiming to identify the outcomes arising from the training on participants’ professional practice. The in-person interviews were conducted by a trained researcher and lasted an average of 60 min. The interviews were recorded, and the content of the interviews was transcribed verbatim.

### 2.4. Data Analysis

#### 2.4.1. Quantitative Analysis

Only fully-completed questionnaires were included in the study. The characterization of the sample was based on a descriptive analysis of the sociodemographic data provided by the participants prior to data collection. When necessary, chi-square tests were performed, with a significance level set at 5%.

All questionnaires underwent descriptive analysis. The analyses were performed using R 3.6.1. For the longitudinal analysis, data from the Likert scale on the self-perception of skills and resources were reorganized into three alternatives, namely: disagree—considered as the sum of the original options totally disagree and disagree; neither agree or disagree—reflecting the original option; and agree—considered as the sum of the original options agree and fully agree. Friedman and, when necessary, Wilcoxon tests were performed using R 3.6.1.

#### 2.4.2. Qualitative Analysis

All qualitative data were subjected to thematic content analysis [20]. The identification of emerging themes and the definition of data analysis categories [21] was carried out through peer triangulation, involving researchers experienced in the processes of qualitative studies. The defined categories and criteria guided the data analysis, which was performed with the aid of NVivo 12 software. 

### 2.5. Ethical Aspects

This study was approved by the Research Ethics Committee of UNIFESP (report numbers 841957 and 2348625). All participants were informed about its objectives and methodologies, with anonymity guaranteed in the dissemination of results and freedom to withdraw at any stage of the research without causing them any harm. This information was presented in the free and informed consent form signed by all who agreed to participate in the research.

## 3. Results

### 3.1. Quantitative Evaluation

Of the 255 training participants who completed at least one of the modules and who agreed to participate in the research, 177 (69.4%) responded to the T1 questionnaires and 47 (18.4%) responded to the T2 questionnaires. At T2, 55.3% of the questionnaires were completed online and 44.7% in person. Only 29 participants completed the questionnaires at all three collection times (T0, T1, and T2).

#### 3.1.1. Post-Training Assessment

Of the 177 participants who completed the T1 questionnaire, 85.6% responded that the training met their expectations, 95.1% responded that the training contributed to the improvement of their professional practices, and 94.3% of the participants said they were satisfied or very satisfied with the topics covered during the training (Table 1). As for the training format, 88% of the participants said they were satisfied or very satisfied and 87.4% said they were motivated or very motivated to disseminate the knowledge. Dissatisfaction rates for these questions ranged from 5.2% to 9.1%. 

#### 3.1.2. Follow-Up Assessment

Out of the 47 participants at T2, 68.1% reported that the training made them think about the practices developed in the service (Table 2). Only one person (2.1%) reported not having noticed any contribution from the training. Regarding the practices covered by the training that were developed in the service, most participants responded that they had taken actions to improve their networking (59.6%) and harm-reduction practices (57.4%). Only one participant (2.1%) reported not having developed any practices in their service. Harm reduction was the topic that most participants thought they were able to convey to their service colleagues (55.3%), followed by networking and collective reflection strategies (both 51.1%).

Regarding the aspects that favored the implementation of practices in the service, most participants selected “personal motivation” (61.7%). The aspect that most compromised practical application was the service team (36.2%), followed by service management (34%). Personal issues were also a factor reported to interfere in professional performance (57.4%).

#### 3.1.3. Longitudinal Assessment

The analysis considering the 29 participants who completed the questionnaires at all three collection times (T0, T1, and T2) showed that the training had a significant effect on the participants’ response patterns (X^2^(2) = 18.97, *p* < 0.005) (Table 3). Wilcoxon analysis showed a significant trend towards greater agreement with the statements between T0 and T1 (*p* < 0.001), with a maintenance in T2 (*p* = 0.001), and with no difference between T1 and T2 (*p* = 0.08). Complementary analyses indicated changes in the pattern of responses between collection times for the axis “Access to information and resources” (X^2^(2) = 22.72; *p* < 0.001) and “Motivation and confidence for practice” (X(2) = 9.09; *p* = 0.011). In both cases, there was greater agreement with the statements at T1 and T2 than at T0 (*p* < 0.03), with no difference between T1 and T2 (*p* > 0.2). No changes were observed in the pattern of responses to the axis “relationship building, communication and horizontality” (X^2^(2) = 2.83; *p* = 0.24), with a tendency for participants to agree with the statements at all times of data collection. 

### 3.2. Qualitative Evaluation

One hundred and fifty-one training participants answered the open questions of the T1 questionnaire, and 35 training participants answered the open questions of the T2 questionnaire. Twenty-eight training participants participated in individual post-training follow-up interviews (T2). Most interviews (71.4%) were carried out at the participant’s service, while 28.6% were carried out at UNIFESP or in neutral public places chosen by the participants. The interview participants were mostly female (71.4%), with a mean age of 47.3 years (SD = 11.8). Most participants held operational (57.1%) and managerial (42.9%) positions, with an average time in the current service of 7.4 years (SD = 7).

The categories defined for the qualitative analysis of the data were organized into three axes: professional, CRR Training, and outcomes (Table 4). The “Professional” axis comprised categories related to the training participant, such as previous training in the AOD area, motivation for being trained and for working in the area, and the characteristics of the participant and the services in which they work. The “CRR Training” area comprised the evaluation of the training by the participants and their perceptions about its contributions to their professional performance. The “Outcomes” axis comprised post-training-related categories, such as the practical application of knowledge in the professional’s routine, network identification and collaboration, and the development of knowledge-dissemination strategies. The detailed analysis is presented below. 

#### 3.2.1. Professional

Participants reported having had little contact with the topic of AOD during graduation, and it was necessary to seek additional training after starting to work in the area. This search for further education follows two paths: first, on the professional’s own initiative, seeking to improve in the area and/or progress in their career; and second, by order of their superiors, who are supposed to allocate part of their human resources to mandatory training. It is noteworthy that many participants reported initially not feeling motivated in respect to this training, taking part due to orders from their superiors or due to an interest in a qualification for career progression. However, these participants reported changes in their attitude during the training process; they to see meaning in their participation and developed a genuine interest in continuing the trainings.

*“First, I just wanted a certificate to add to my qualifications, but when I went to the first meeting I felt something different. I said to myself, ‘There’s something innovative here, something that will add to my experience.’”*

(Category Motivation; Interview T2—Education Manager)

Most of the professionals were working in nonspecialized services on AOD, but dealt with this issue crosswise their work routine, although they reported not have any related training that prepared them to do so. Moreover, most of the services have a high turnover of staff and a shortage of human resources, resulting in a considerable workload. Participants also reported a rigid hierarchical organization in most services, so that even the leading professionals had little autonomy. The participants stated that work overload and a lack of autonomy led them to a state of exhaustion and demotivation, with some individuals needing treatment for mental disorders such as depression and anxiety.

*“The problem with public agencies is this, replacing professionals who are permanently absent is hard work!”*

(Category Characteristics; Interview T2, Public Security Technician)

There are, however, some reports of well-integrated teams, able to develop interprofessional work with good outcomes for service users. Most of these reports came from service managers, who reported that after training, they organized their team with the aim of establishing a horizontal relationship. These managers stressed that changes were only possible because they occupy a management position.

*“This training was valuable to me because I’m in a managerial position, so I can create a project and ask for collaboration from the team. Those who took the training who were not in my position cannot just go to the manager and say ‘this is what I’ll do!’”*

(Category Characteristics; Interview T2, Public Security Manager)

#### 3.2.2. CRR Training

The training was well evaluated by the participants who valued interprofessional contact, horizontalized discussions, and practical activities. Only one participant reported not being interested in the reflective approach of the training, preferring traditional expository classes. The participants’ main criticisms were related to logistical issues, such as class hours and the location of the training activities.

*“Maybe because of the way in which some subjects were approached, with a certain freedom, and a non-judgmental tone, each person could say what they thought and discuss the answer together...”*

(Category Evaluation; Interview T2, Health Technician)

The major contributions of the training were related to better understanding of issues related to substance use disorders and a change in the view of AOD users. Participants also reported a reduction in their perception of the stigma associated with substance users, and an increase in the feeling of “not being alone”.

*“It made me look with fresh eyes and reconsider some stigmas.”*

(Category Contributions; Questionnaire T1)

*“I didn’t understand the complexity of addiction, what an addict was, and the training made me change my point of view.”*

(Category Contributions; Interview T2, Public Security Manager)

#### 3.2.3. Outcomes

After the training, participants reported reflecting on their professional performance, prompting a process of reviewing attitudes and practices. Again, differences emerged between operational and managerial staff. Only the managers were able to develop projects or perform systemic changes in their services, while the professionals at an operational level ended up focusing on personal actions, with changes in their individual practices. In the T2 questionnaire, training participants mentioned financial issues as an important factor for not implementing changes in their services. In the interviews at T2, participants further expressed what they were able to achieve. Many participants reported difficulties in receiving support from their teams for the implementation of innovations, since most other service professionals had not completed the training processes. Consequently, their teams would not have the same knowledge or motivation to change. Both operational staff and managers agreed that management support is crucial for the development of systemic changes, since these positions bring autonomy.

*“I created a project with several activities, there were sports, cinema, karaoke, art classes… the first result I had was with an extremely aggressive intern who were in trouble every week. In sport he didn’t play, he fought; he didn’t like singing, he wasn’t going to watch movies, but he was the first intern to paint a picture! (...) It got results!”*

(Category Practical Application; Interview T2, Public Security Manager)

*“I even did some activities with my patients. I still find myself saying some things I learned in the training...”*

(Category Practical Application; Interview T2—Health Technician)

In general, the changes implemented were aimed at meeting the multiple needs and demands of the service users, which involved knowledge and interaction within a network. Participants reported an increase in their understanding of the concept of working networks, which included within- and between-service interrelationships for caring individuals. Many of the implemented changes required intense coordination between networks, with the development of projects and actions that required significant interprofessional working. However, several reports showed the existence of fragile networks which were poorly-coordinated and centered on individuals rather than institutions.

*“I ended up knowing other sources that I can go to for help. There were some situations that I didn’t even know these places existed to help.”*

(Category Network Relationship; Interview T2, Education Manager)

*“We made friends within the groups, introduced ourselves, explained where we were from, so the network developed in that way. The network is personal(...)”*

(Category Network Relationship; Interview T2, Social Worker)

In the T1 questionnaire, participants reported that they were unable to develop related activities due to a lack of management support and interest from their service teams. During the follow-up interviews at T2, participants reported using team meetings to reflect collectively and share knowledge. A few participants also reported initiatives to include the new knowledge in internal training programs within the services, and some started thematic study groups.

*“Look, we have meetings where we talk about doubts, approaches, discuss cases...”*

(Category Knowledge Dissemination; Interview T2, Justice Professional)

## 4. Discussion

The longitudinal evaluation of the CRR-DU trainings showed that they were well received by the participants, who positively evaluated the thematic and pedagogical strategies used during the different modules. According to participants’ views, the trainings seem to have promoted changes in ideas and attitudes, encouraging reflective thinking of their professional practice and the possibilities for improvement. Both quantitative and qualitative results showed that the main outcomes of the trainings were related to a greater understanding of the multifactorial basis of AOD, the reduction in stigma, a change in how students perceive drug users, and the expansion of the repertoire of service networks. The biopsychosocial approach of the trainings with their horizontal, reflective nature; the practical interprofessional approach with heterogeneous classes; and the field experiences provided in the experiential modules seem to have been crucial in facilitating the achieved outcomes.

The biopsychosocial approach [22] allowed the training participants to develop a greater understanding of the complexities related to the harmful use and dependence on AOD, demystifying concepts and theoretically grounding key points previously not understood. This understanding was perceived as an important factor for promoting changes on how the participants perceived AOD use and users. After the training, participants reported more welcoming attitudes, less stigmatization, and increased focus on the range of needs of the service users. Smith (2020) [23] argued that the biopsychosocial approach to drug use allows the development of an understanding of its multifactorial basis, both internal and external, which interact with each other in the process of harmful use and substance dependence. In the same way, the different treatments also interact with each other, enhancing or hindering their results. It is, therefore, necessary that treatments have multimodal and multilevel approaches and consider the different issues related to substance use and the different consequences of the interventions used, including the indirect effects of the treatment itself [23]. Thus, biopsychosocial knowledge favors a reflection on the best strategies to approach issues related to substance use, considering the individual in an integral and humanized way, in all their complexity [24], as was observed in the experience described herein.

Interprofessional education, in turn, has the potential to promote the development of collaborative practices, which can result in better outcomes for patients [7,8,9,25]. In 2009, Mann et al. [26] observed an increase in communication between professionals from different areas, with more confidence, assertiveness, and respect after the implementation of an interprofessional curriculum in a cancer treatment center [26]. Muzyk et al. (2019) [27] observed improvements in the attitudes, interprofessional collaboration, and decision making of health students towards the treatment of patients with substance use disorders [27]. More recently, Gainey et al. (2022) [28] reported that an interprofessional SBIRT training provided an increase in knowledge, skills, and confidence to practice in health students [28]. The interprofessional experiences during the training promoted the interaction between professionals in different sectors, which may help to increase their perception of the breadth of the network in which they are a part of and reduced the feeling of isolation. This approach allowed them to understand and discuss the same theme from different perspectives, promoting the understanding and appreciation of the role of the other, in addition to stimulating the development of partnerships and collaborations.

The experiential modules enabled the participants to put into practice what was discussed in the classroom, beyond the settings of the services in which they worked. Participants had the opportunity to experience different realities within the same network, promoting the demystification of concepts and reducing the stigma associated with other services, in addition to the development of practical skills, such as communication and interprofessional working. These experiences seem to have been of great value during the learning process, enabling the integration of knowledge that had been built up throughout the trainings. Pidd (2010) [10] reported that the approximation of theoretical knowledge with real life is fundamental for the training of professionals working in the field of AOD and encourages the development of practical skills that can be useful in their routines [10].

With respect to stigma, the training proposed to work through two of the three recommended approaches for the reduction in stigma: education, developed in the theoretical modules; and contact [29], provided in the experiential modules. The reports of the participants showed that this strategy provided a chance to reflect on their beliefs and attitudes and encourage a change in their position towards issues related to the use of AOD.

The individual interviews indicated that the training participants were very aware of their increased understanding on AOD and reported changes in their individual performance as a result of the trainings, but they faced difficulties in implementing systemic changes in their workplaces. These difficulties were quantitatively and qualitatively associated with the lack of support from management and service teams, in addition to a lack of time and work overload. Several other authors encountered the same difficulties with respect to the process of transforming theoretical knowledge into practice [26,30,31] and some strategies for overcoming this have been proposed. Christie et al. (2013) [31] observed in their study that training participants expressed an interest in having a post-training follow-up. As was observed in this study, better outcomes were achieved when leaders were involved in the change process, which is also recommended by several other authors [26,32,33]. Sebastian et al. (2020) [34] highlight the importance of adapting the training content and interventions to be implemented in the professional’s routine, in a way to make them viable to real-life adoption [34]. Greenhalgh et al. (2004) [35] suggest that it is possible to have better results by training entire teams, rather than individuals alone. They also argue that a flexible and adaptive organizational structure which embraces innovation facilitates its implementation, and that the continuous support and commitment of managers to projects and development, communication, intra-organizational alignment (everyone knows what they are doing and why they are doing it), and feedback on the implementation process are extremely important. Finally, they report that the more complex the innovation, the more important is the involvement of partner networks in the process [35].

Looking to increasing the potential of improvement in the performance of the professionals working in the area of AOD, we provide four core recommendations for future trainings in the area: (1) working with the biopsychosocial approach and interprofessional education formats as a way to reinforce the need to treat substance users in an expanded and humanized way; (2) to align the training content with the needs of its participants, which can be established by conducting previous assessments of needs and demands; (3) to use educational methodologies that encourage the reflection and the exchange between peers of their real life practice, promoting meaningful learning and contributing to the incorporation of constructed knowledge and possible transformation into practice by the professionals; (4) to involve key stakeholders, leaders, and service teams, which seems to be crucial for promoting systemic changes. Additionally, offering post-training support to help during the initial stages of implementing changes can also favor the implementation of improvements in the performance of the professionals working in the area.

As a longitudinal study, this work is distinguished by the use of convergent mixed methods, which allowed both the comparison of results over time (the beginning of the training, at the end of the training, and one year after its conclusion) and the triangulation of results by quantitative and qualitative methods [15]. Altogether, this approach favored a global view of the trainings offered and their outcomes, and an in-depth analysis that allowed an understanding of the reasons for the observed phenomena.

Within the qualitative data, we also highlight the performance of the triangulation of the results with respect to the techniques [36,37], comparing the data from the questionnaires and the interviews with the participants. The use of mixed methods strengthens the study, since all the data converged to the same results in a complementary way [38,39]. This type of approach proved to be appropriate for the longitudinal assessment of the training, allowing objective analyses that provide a global view of the trainings offered and their outcomes, and in-depth analyses that allow an understanding of the reasons for the observed phenomena.

This study may have a response bias that should be considered. Since not all invited participants agreed to participate in the study, we must consider the possibility that the participants who accepted may have characteristics that could influence the results. The low longitudinal response rate is another limitation of this study, and we were not able to carry out advanced statistical analyses. The literature shows that online surveys carried out with health professionals do not usually obtain large levels of return, especially when financial or other incentives are not offered [18,19,40,41,42], with an average return of 20% being reported in different studies [19,43,44]. It is important to consider the feasibility of using exclusively quantitative analyses in longitudinal assessments, since it is usually difficult to obtain adequate rates of return from the participants to carry out the intended statistical analyses. We believe it is important to consider the use of in-depth assessments to access possible long-term outcomes in the routine of professionals.

## 5. Conclusions

The trainings offered seem to have promoted the understanding of the complexity of the issues related to substance use and dependence, produced a reduction in stigma, changed the way that the participants perceived drug users and services related, and helped to expand skills and knowledge. The main characteristics related to these outcomes seem to have been the interprofessional and biopsychosocial approach and field activities. Although participants reported a change in attitude towards the topic, they also reported facing difficulties in implementing systemic changes in their services, actions that seem to have been carried out only by professionals in management positions. To overcome these difficulties, it is recommended that in future training there is greater involvement of managers and service teams. It is also recommended that AOD training assessments consider long-term outcomes, seeking to identify whether the knowledge built during the training is converted into positive practices in the professional’s routine.

## Figures and Tables

**Table 1:** Post-training evaluation reported by 177 participants in T1 questionnaire.

Question	Response	N	%
Did the training meet your expectations?	No	25	14.4
	Yes	149	85.6
Did the training contributed to the improvement of your professional practice?	No	8	4.8
	Yes	157	95.2
How satisfied are you with the topics covered during the training?	Very unsatisfied	0	0.0
	Unsatisfied	9	5.2
	Indifferent	1	0.6
	Satisfied	95	54.6
	Very satisfied	69	39.7
How satisfied are you with the training format? (Theoretical discussions, practical activities, etc.)	Very unsatisfied	0	0.0
	Unsatisfied	16	9.1
	Indifferent	5	2.9
	Satisfied	98	56.0
	Very satisfied	56	32.0
How motivated are you to disseminate the knowledge acquired during the training?	Unmotivated	2	1.1
	Little motivated	13	7.4
	Indifferent	7	4.0
	Motivated	104	59.4
	Very motivated	49	28.0

**Table 2:** Post-training outcomes and factors related to professional performance reported by 47 participants in the T2 questionnaire.

**Outcomes**	**Response**	**N**	**%**
Training contributions to practice	Reflections on practices	32	68.1
	Networking	29	61.7
	Relationship with the user	25	53.2
	Identifying networks	21	44.7
	Stigma reduction	19	40.4
	Relationship with users’ family members	16	34.0
	Intra-team networking	15	31.9
	Diagnosis and intervention	13	27.7
	None	01	2.1
Practical application of knowledge	Networking	28	59.6
	Damage Reduction	27	57.4
	Prevention strategies	24	51.1
	Brief Intervention	21	44.7
	Interdisciplinary work	21	44.7
	Screening Instruments	09	19.1
	Community treatment	06	12.8
	None	01	2.1
Knowledge dissemination	Damage Reduction	26	55.3
	Networking	24	51.1
	Collective reflection	24	51.1
	Prevention strategies	22	46.8
	Brief Interventions	16	34.0
	Screening Instruments	10	21.3
	None	04	8.5
**Factors**	**Response**	**N**	**%**
Favorable to implementation	Personal motivation	29	61.7
	Relationship with the user	23	48.9
	Networking	20	42.6
	Service team	18	38.3
	Service management	16	34.0
	Relationship with the community	15	31.9
Unfavorable to implementation	Service team	17	36.2
	Service management	16	34.0
	Networking	10	21.3
	Relationship with user	05	10.6
	Relationship with the community	04	8.5
	Personal motivation	00	0.0
Other factors related to professional performance	Personal issues	27	57.4
	Other Capabilities	18	38.3
	Public policy changes	15	31.9
	Management changes	06	12.8
	None	03	6.4

**Table 3:** Self-perception of 29 participants about their skills and resources to work in AOD, answered in T0, T1 and T2. Likert scale presented as percentage and confidence interval, organized in three axis: (A) Access to information and resources; (B) Motivation and confidence to practice; (C) Relationship building, communication, and horizontality.

	Affirmatives		Disagree % [CI]	Indifferent % [CI]	Agree % [CI]
**A**	I have access to information	T0	37.9 [22.7–56.0]	3.4 [0.06–1.7]	58.6 [40.7–74.5]
		T1	3.4 [0.06–1.7]	6.9 [1.9–22.0]	89.7 [73.6–96.4]
		T2	13.8 [ 5.5–30.6]	6.9 [1.9–22.0]	79.3 [61.6–90.2]
	I have access to resources	T0	48.3 [31.4–65.6]	10.3 [3.6–26.4]	41.4 [25.5–59.3]
		T1	10.3 [3.6–26.4]	13.8 [5.5–30.6]	75.9 [57.9–87.8]
		T2	37.9 [22.7–56.0]	13.8 [5.5–30.6]	48.3 [31.4–65.6]
	I use search results to do my work	T0	41.4 [25.5–59.3]	17.2 [7.6–34.5]	41.4 [25.5–59.3]
		T1	10.3 [3.6–26.4]	24.1 [12.2–42.1]	65.5 [47.3–80.1]
		T2	13.8 [ 5.5–30.6]	20.7 [9.8–38.4]	65.5 [47.3–80.1]
	I have access to tools to identify users’ needs	T0	50.0 [32.6–67.4]	25.0 [12.7–43.4]	25.0 [12.7–43.4]
		T1	21.4 [10.2–39.5]	10.7 [3.7–27.2]	67.9 [49.3–82.1]
		T2	34.5 [19.9–52.7]	13.8 [ 5.5–30.6]	51.7 [34.4–68.6]
	I have access to tools to evaluate results	T0	60.7 [42.4–76.4]	10.7 [3.7–27.2]	28.6 [15.3–47.1]
		T1	21.4 [10.2–39.5]	14.3 [5.7–31.5]	64.3 [45.8–79.3]
		T2	24.1 [12.2–42.1]	27.6 [14.7–45.7]	48.3 [31.4–65.6]
	I have access to news from my area	T0	25.0 [12.7–43.4]	21.4 [10.2–39.5]	53.6 [35.8–70.5]
		T1	10.3 [3.6–26.4]	10.3 [3.6–26.4]	79.3 [61.6–90.2]
		T2	17.2 [7.6–34.5]	10.3 [3.6–26.4]	72.4 [54.3–85.3]
**B**	I know what to do to help the users	T0	37.9 [22.7–56.0]	20.7 [9.8–38.4]	41.4 [25.5–59.3]
		T1	6.9 [1.9–22.0]	3.4 [0.06–1.7]	89.7 [73.6–96.4]
		T2	3.4 [0.06–1.7]	13.8 [5.5–30.6]	82.8 [65.5–92.4]
	I can monitor the results	T0	28.6 [15.3–47.1]	17.9 [7.9–35.6]	53.6 [35.8–70.5]
		T1	17.9 [7.9–35.6]	14.3 [5.7–31.5]	67.9 [49.3–82.1]
		T2	17.2 [7.6–34.5]	20.7 [9.8–38.4]	62.1 [44.0–77.3]
	I know When and how to make referrals	T0	25.0 [12.7–43.4]	14.3 [5.7–31.5]	60.7 [42.4–76.4]
		T1	0.0	14.3 [5.7–31.5]	85.7 [68.5–94.3]
		T2	6.9 [1.9–22.0]	13.8 [5.5–30.6]	79.3 [61.6–90.2]
	I feel motivated to do my work	T0	10.7 [3.7–27.2]	21.4 [10.2–39.5]	67.9 [49.3–82.1]
		T1	3.6 [0.6–17.7]	7.1 [2.0–22.6]	89.3 [72.8–96.3]
		T2	0.0	10.3 [3.6–26.4]	89.7 [73.6–96.4]
	I trust my ability to do my work	T0	3.7 [0.7–18.3]	7.4 [2.1–23.4]	88.9 [71.9–96.2]
		T1	0.0	3.4 [0.06–1.7]	96.6 [82.8–99.4]
		T2	3.4 [0.06–1.7]	0.0	96.6 [82.8–99.4]
**C**	Help the users to identify their resources	T0	24.1 [12.2–42.1]	6.9 [1.9–22.0]	69.0 [50.7–82.7]
		T1	3.4 [0.06–1.7]	13.8 [5.5–30.6]	82.8 [65.5–92.4]
		T2	6.9 [1.9–22.0]	20.7 [9.8–38.4]	72.4 [54.3–85.3]
	I discuss cases in my team meetings	T0	17.9 [7.9–35.6]	14.3 [5.7–31.5]	67.9 [49.3–82.1]
		T1	6.9 [1.9–22.0]	6.9 [1.9–22.0]	86.2 [69.4–94.5]
		T2	10.3 [3.6–26.4]	13.8 [5.5–30.6]	75.9 [57.9–87.8]
	I consult other colleagues	T0	3.6 [0.6–17.7]	3.6 [0.6–17.7]	92.9 [77.3–98.0]
		T1	3.6 [0.6–17.7]	0.0	96.4 [82.3–99.4]
		T2	3.4 [0.06–1.7]	3.4 [0.06–1.7]	93.1 [78.0–98.1]
	I care about the users’ preferences	T0	7.1 [2.0–22.6]	7.1 [2.0–22.6]	85.7 [68.5–94.3]
		T1	0.0	3.6 [0.6–17.7]	96.4 [82.3–99.4]
		T2	3.6 [0.6–17.7]	3.6 [0.6–17.7]	92.9 [77.3–98.0]
	I understand the feelings and behaviors of the users	T0	0.0	3.6 [0.6–17.7]	96.4 [82.3–99.4]
		T1	3.4 [0.06–1.7]	0.0	96.6 [82.8–99.4]
		T2	10.3 [3.6–26.4]	0.0	89.7 [73.6–96.4]
	I understand the feelings and behaviors of the user’s family members	T0	3.7 [0.7–18.3]	7.4 [2.1–23.4]	88.9 [71.9–96.2]
		T1	0.0	0.0	100.0
		T2	6.9 [1.9–22.0]	0.0	93.1 [78.0–98.1]

**Table 4:** Descriptive scheme of qualitative analysis. On top are the three axis on which the categories were organized, followed by the respective categories of analysis and its descriptions.

PROFESSIONAL	CRR TRAINING	OUTCOMES
**Training on AOD** History of training in AOD prior to working in the area.	**Evaluation** Participants’ evaluation of the CRR training offered.	**Practical applications** Application of knowledge in the profefessionals’ work routine.
**Motivation** Motivation for being trained and for working in AOD area.	**Contributions** Participants’ perceptions about the training contributions to their professional performance.	**Network relationship** Identification and working collaboration within the services network.
**Characteristics** Characteristics of the participants of the study and the services in which they work.		**Knowledge dissemination** Strategies to dissemination of the training content.

## Data Availability

The data presented in this study are available on request from the corresponding author. The data are not publicly available due to ethical reasons.
